# Dual‐PKS Cluster for Biosynthesis of a Light‐Induced Secondary Metabolite Found from Genome Sequencing of *Hyphodiscus hymeniophilus* Fungus

**DOI:** 10.1002/cbic.201900689

**Published:** 2020-04-21

**Authors:** Glenna J. Kramer, Sheila Pimentel‐Elardo, Justin R. Nodwell

**Affiliations:** ^1^ Department of Biochemistry University of Toronto MaRS Centre, West Tower 661 University Avenue Toronto ON M5G 1M1 Canada

**Keywords:** genomics, natural products, polyketides

## Abstract

Filamentous fungi are known producers of important secondary metabolites. In spite of this, the majority of these organisms have not been studied at the genome level, leaving many of the bioactive molecules they produce undiscovered. In this study, we explore the secondary metabolite potential of an understudied fungus, *Hyphodiscus hymeniophilus*. By sequencing and assembling the first genome from this genus, we show that this fungus has genes for at least 20 natural products and that many of these products are likely novel. One of these metabolites is identified: a new, red‐pigmented member of the azaphilone class, hyphodiscorubrin. We show that this metabolite is only produced when the fungus is grown in the light. Furthermore, the biosynthetic gene cluster of hyphodiscorubrin is identified though homology to other known azaphilone producing clusters.

## Graphical abstract

**Red/light metabolite**: The fungus *H. hymeniophilus* produces a red, light‐inducible secondary metabolite. This molecule, hyphodiscorubrin, is shown to be a novel, azaphilone‐like molecule. Whole‐genome sequencing revealed the potential for natural product production, along with the identification of a putative dual‐PKS gene cluster for hyphodiscorubrin production.

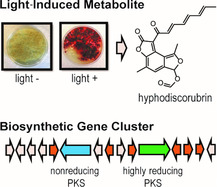

Filamentous fungi are part of a diverse, ubiquitous and understudied kingdom of organisms. Less than 120 000 species of fungi have been identified, even though estimates suggest that there are over 2 million species of fungi globally.^1^ Fungal secondary metabolites are of extraordinary value. However, as many genera of fungi, including *Hyphodiscus,* have not been thoroughly studied for secondary metabolite production, the full chemical diversity of this kingdom remains unknown.^2^ Known examples of fungal secondary metabolites include some of the most important drugs in history including the antibiotic penicillin and lovastatin, used clinically to lower cholesterol levels.^3^

Bioinformatic analysis suggests that many fungal species produce well over 20 distinct natural products, many of them unknown.^4^ Indeed, we recently reported that the genome of *Cordyceps militaris* has biosynthetic genes for at least 34 natural products.^5^ One roadblock to characterizing these molecules may be that their genes are not expressed under typical laboratory conditions.^6^ Fungi live in complex environmental niches and likely produce secondary metabolites in response to their surroundings, allowing them to modulate their environment and compete with other microbes.^7^ As is the case with natural product‐producing bacteria, the specific cues that trigger production of many natural products are unknown and must be bypassed in the laboratory.^8^, ^9^

In this study, we focus on the fungal species *Hyphodiscus hymeniophilus* ATCC 34498. This fungal species is most closely related to those in the Leotiomycete family and is considered an anamorph of the strain *Catenulifera rhodogena*. (Figure 1A, Table S1 in the Supporting Information).^10^ Several strains of this fungus have been characterized throughout Europe, often times noted as a reddish fungus growing on decaying wood.^11^ This particular ATCC strain was isolated from an insect cadaver, and has remained understudied, although it is an accessible fungal specimen.

**Figure 1:**
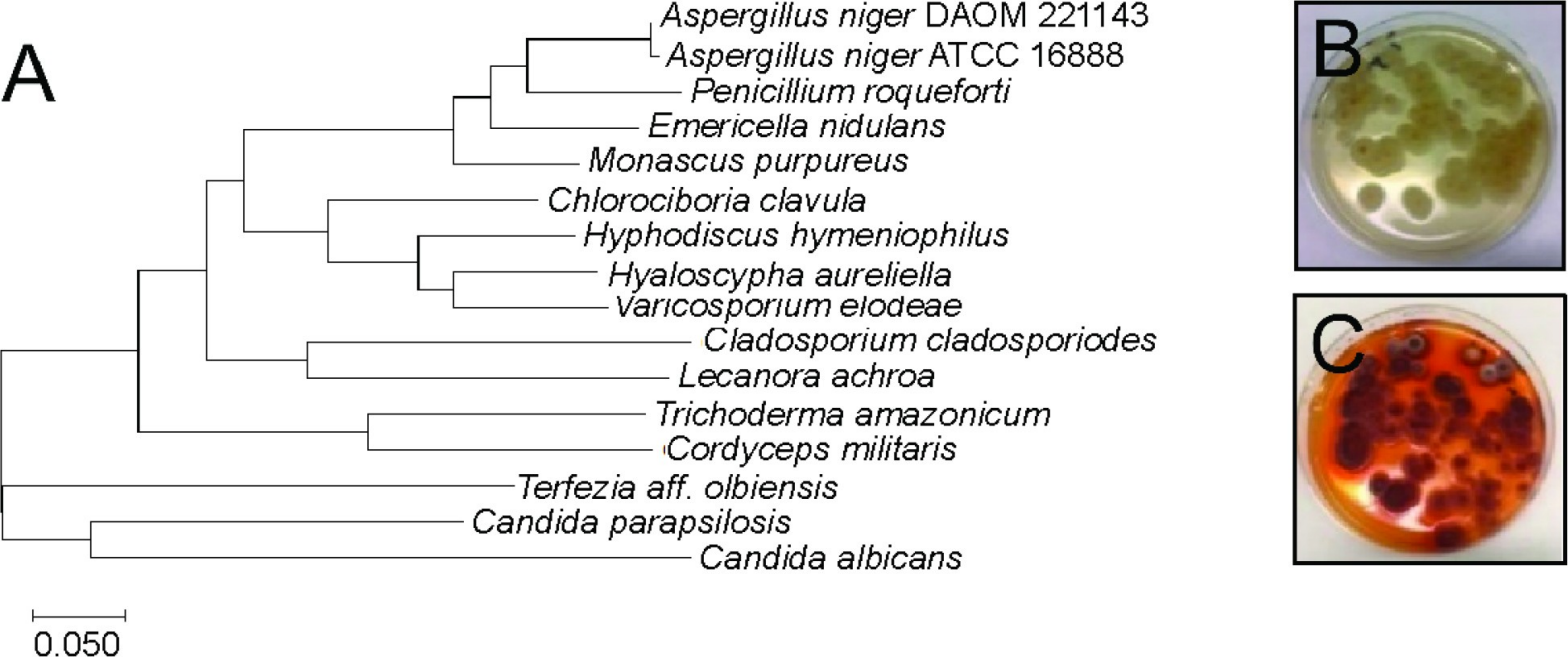
A) Phylogenetic tree of fungal ITS regions showing that *Hyphodiscus* clusters with fungi from the Leotiomycete family. B) *Hyphodiscus* grown in the dark. C) *Hyphodiscus* after being exposed to 12‐hour light/dark cycles.

Herein, we report the first genome sequence of *Hyphodiscus hymeniophilus,* describe the natural product potential of this organism, and show that many metabolites this species may have the capacity to produce are unknown. We identify one of these natural products, hyphodiscorubrin, showing that the biosynthesis of this novel, red‐pigmented, azaphilone‐like molecule is triggered by light. Additionally, we identify a dual‐PKS gene cluster, a cluster that contains two polyketide synthase genes, that is likely responsible for the biosynthesis of hyphodiscorubrin. Our work further demonstrates the remarkable chemical potential of fungi and supports the notion that a spectrum of approaches and strategies will be necessary to express, purify and characterize fungal secondary metabolites.

During a broad screen for fungal secondary metabolites, we generated small molecule extracts of *Hyphodiscus*. These extracts were screened for antibacterial activity against *E. coli* and *B. subtilis*, antifungal activity against *Saccharomyces cerevisiae* and insecticidal activity against *Drosophila melanogaster* larvae. Simultaneously, these extracts were analysed for unique or differentially expressed metabolites via liquid spectrometry mass spectroscopy (LCMS). *Hyphodiscus* extracts did not exhibit bioactivity against any of these systems, however, this specimen attracted our attention as it unexpectedly produced a reddish metabolite when grown in 12‐ hour light/dark cycles, but not when the fungus was grown solely in the dark (Figure 1B, C).

By growing *Hyphodiscus* in the dark for two weeks followed by treating the mycelia with 12‐hour light/dark cycles for 4 days, enough of the metabolite was produced for purification of a single reddish‐orange metabolite. This metabolite was purified using a column chromatography approach with a mobile phase containing formic acid to give hyphodiscorubrin, with a UV max of 474 nm and a parent ion at *m/z* 367.1179 [M+H]+ (calcd. exact mass 367.1181), corresponding to a molecular formula of C_21_H_18_O_6_ (Figure S1). We did observe that this metabolite was chemically reactive during purification. Attempts to purify this product using a mobile phase that contained an amine (ammonium acetate) resulted in a smear of various orange and red molecules over several fractions during the purification. Further analysis of the major product of the ammonium acetate containing purification approach indicates that this metabolite has a reddish‐pink colour, a UV max of 540 nm and 366.1351 [*M*+H]+ (calcd. exact mass 366.1341), corresponding to a molecular formula of C_21_H_19_NO_5_ (Figure S2). This is consistent with an azaphilone‐like molecule as molecules from this class have been shown to undergo spontaneous reactions with amines in which an oxygen heteroatom is replaced by a nitrogen.^12^, ^13^ A reaction of this sort is supported through a change in mass and a shift in the UV spectrum, similar to what we observe. In the case of hyphodiscorubrin, the amine‐containing variant appeared to be produced at a low yield and was unstable, therefore we were unable to isolate enough for a complete characterization. Therefore, our structural characterization is conducted on hyphodiscorubrin which has been purified using a mobile phase containing formic acid. Experimental details on the purification of hyphodiscorubrin are given in the Supporting Information.

Further analysis of hyphodiscorubrin confirmed a chemical formula of C_21_H_18_O_6_ by high resolution mass spectrometry (HRMS‐ESI) suggesting 13 degrees of unsaturation. ^1^H NMR data showed the presence of 18 protons with three peaks integrating for three protons and nine peaks integrating for one proton (Figure 2). The ^13^C NMR showed the presence of 21 carbons. The HSQC‐DEPT spectrum showed a correlation between every proton peak and a carbon peak, suggesting all of the protons in the molecule are attached directly to a carbon and all of the protons are either CH or CH_3_. There are nine remaining carbons that are not directly attached to a proton.

**Figure 2:**
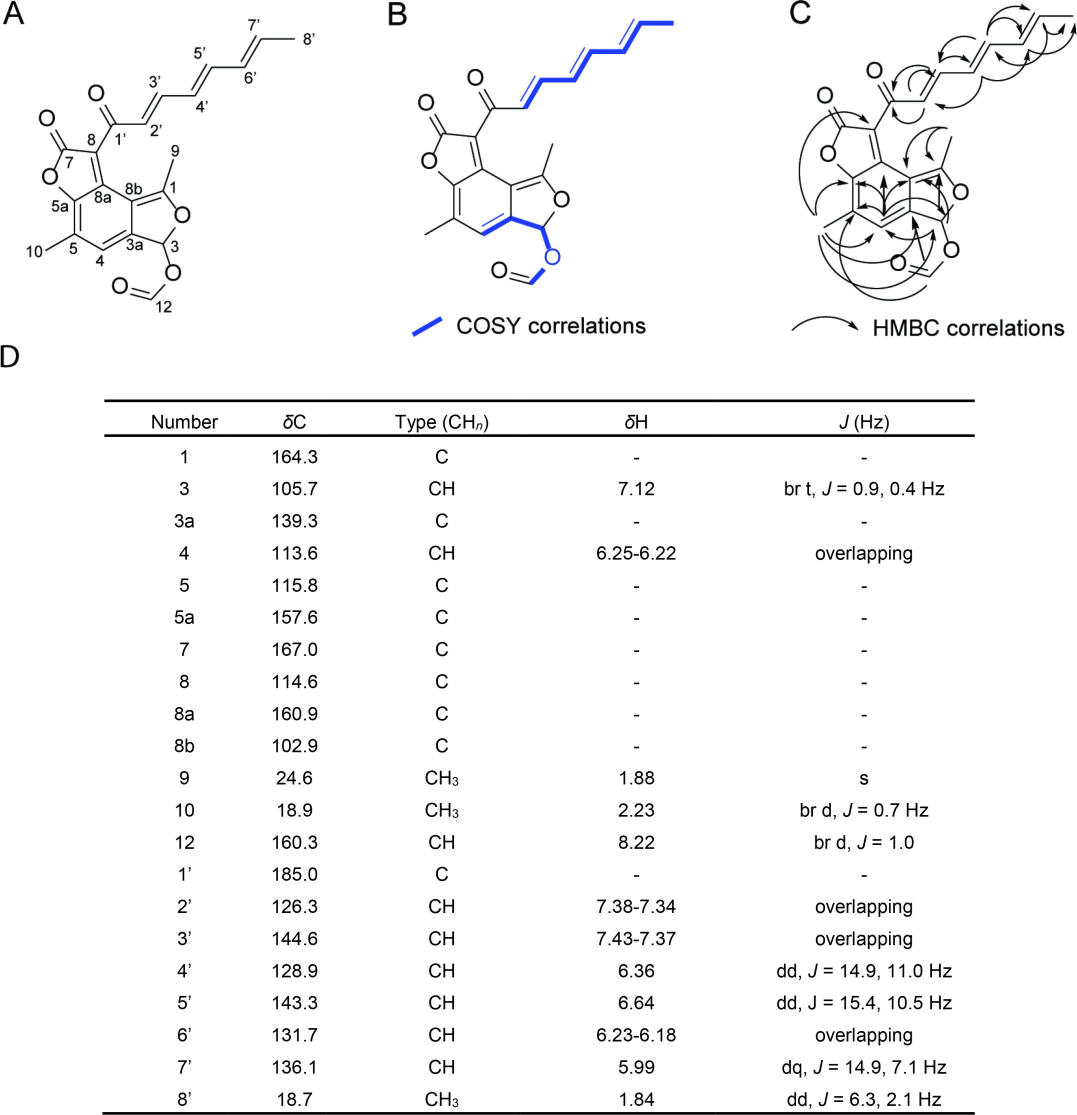
^1^H and ^13^C NMR data for hyphodiscorubrin collected in CDCl_3_. A) Proposed structure with atoms numbered. B) COSY correlations. C) HMBC correlations; independent and long‐range HMBC experiments were performed. D) Table of measured shifts from acquired NMR spectra.

The presence of a seven carbon chain (C‐2’ through C‐8’) is supported by COSY and HMBC correlations (Figure 2B, C). This unsaturated hydrocarbon chain is shown to be attached to C‐1’ through an HMBC correlation. C‐1’ is likely to be a conjugated carbonyl to have the chemical shift of *δ*=185.0. Aside from this hydrocarbon chain, there are two additional methyl groups present, comprised of C‐9 and C‐10. Additionally, C‐12 exhibits chemical shifts (*δ*_c_ 160.3 and *δ*_H_ 8.22) suggesting a chemical environment similar to that of a formate group. The remaining carbons in this molecule must be part of a highly conjugated system.

Using HMBC NMR, long‐range HMBC correlations, and HRMS‐ESI, we proposed a structure for hyphodiscorubrin (Figure 2). There are no protons in this structure which have a correlation with C‐7, even in a long‐range HMBC experiment, suggesting that this atom is in a region of the molecule isolated from protons. With a carbon shift of *δ*=167.0, C‐7 is likely to be a carbon in an ester. A COSY correlation between the protons on C‐12 and C‐3 suggests that this formate group is adjacent to C‐3. HMBC correlations suggest that the methyl group of C‐10 is near C‐5a and C‐4 and the methyl group of C‐9 is near C‐1 and C‐8b. Further HMBC correlations and long‐range HMBC correlations were helpful in piecing these atoms together into a highly conjugated three ring structure. During HRMS acquisition it was noted that there was source fragmentation of this molecule giving predominant peaks at 177.0537, 205.0490, and 273.0391 in positive mode. These fragments are supported by analysing the proposed structure using the CFM‐ID fragmentation modelling tool.^14^ NMR and HRMS‐ESI spectra are available in Figures S3‐S9.

In parallel, we sequenced the fungus using Pac Bio long‐read sequencing (180x coverage) and assembled the data into 23 contigs ranging in size from 61,228 bp to 2.86 Mbp. Of these, 18 had characteristic telomeric repeats of (TTAGGG)_*n*_ and (CCCTAA)_*n*_ on both ends suggesting that they are completely sequenced chromosomes. The smallest contig, number 23, (Hyd_23) was identified as mitochondrial DNA.^15^, ^16^ Hyd_23 does not have telomeres on the ends, however it is not apparent whether the DNA is circular.

We annotated this genome using MAKER showing that Hyphodiscus contains 8224 genes (Table S2).^17^ Analysis of the natural product potential of this fungus using FungiSMASH suggested that this organism can produce at least 24 distinct secondary metabolites from at least five distinct classes (Table 1).^18^ Interestingly 18 of these secondary metabolites do not have homology with any characterized cluster suggesting that they are potentially novel. Those clusters with homology to known biosynthetic gene clusters have similarities with clusters that have been described to produce pestheic acid, ACT‐toxin, sodarin, aflatoxin and azanigerone.

**Table 1:** Secondary metabolites predicted in the nuclear DNA of the genome of *H. hymeniophilus*.

Contig	Cluster	Type	Most similar
	number		known cluster
Hyd_1	1.1	T1PKS	–
Hyd_1	1.2	terpene	–
Hyd_1	1.3	NRPS‐like	–
Hyd_3	3.1	T3PKS	–
Hyd_5	5.1	terpene	–
Hyd_5	5.2	T1PKS	pestheic acid
Hyd_7	7.1	NRPS	–
Hyd_7	7.2	NRPS−T1PKS	–
Hyd_7	7.3	T1PKS	–
Hyd_9	9.1	fungal RiPP	–
Hyd_10	10.1	NRPS	–
Hyd_10	10.2	β‐lactone	ACT toxin
Hyd_10	10.3	NRPS	‐
Hyd_11	11.1	NRPS	sodarin
Hyd_11	11.2	T1PKS	‐
Hyd_15	15.1	β‐lactone NRPS	–
Hyd_15	15.2	T1PKS	azanigerone
Hyd_16	16.1	NRPS	–
Hyd_17	17.1	terpene	–
Hyd _17	17.2	NRPS	–
Hyd_17	17.3	T1PKS	–
Hyd_17	17.4	T1PKS	ACT toxin
Hyd_19	19.1	NRPS	–
Hyd_20	20.1	T1PKS	aflatoxin

Contig 20 (Hyd_20) appeared to be an end‐to‐end chromosome of only 205,279 bp and contained one gene cluster (20.1) exhibiting homology to an aflatoxin producing cluster with the polyketide synthase, D0Z07_9324, having 99 % coverage and 50 % identity with aflatoxin biosynthesis protein C from *Aspergillus flavus* NRRL3357. Bioactivity screens of extracts from this fungus against *Drosophila* larvae did not suggest toxicity of these extracts, leading us to believe that this cluster is likely not expressed during laboratory culture. Fungi are known to acquire entire accessory chromosomes for a fitness advantage, so it would be reasonable to believe that this chromosome could be for that purpose.^19^

The genome of *Hyphodiscus,* reveals a dual‐PKS cluster with homology to the azanigerone producing cluster from *Aspergillus niger* (cluster 15.2, Table 1).^20^ Azanigerone belongs to the azaphilone class of fungal metabolites which are often orange or red in colour. As azaphilones have structural similarities to hyphodiscorubrin, it occurred to us that these genes might account for the coloured pigmentation of light‐grown *Hyphodiscus*.^13^

Several other similar dual‐PKS clusters have been characterized in fungi as known producers of azaphilone‐like molecules including azanigerone, asperfurone and chaetoviridin (Figure 3A, B, E).^21^, ^22^, ^23^ In these dual PKS clusters, the core is comprised of both a highly reducing (HR) and a non‐reducing (NR) PKS. In the case of hyphodiscorubrin, it would be reasonable that the non‐reducing PKS (D0Z07_8183) could be partially responsible to the biosynthesis of the bicyclic core of the metabolite, similar to what has been described in the biosynthesis of the bicyclic core of asperfuranone^21^ (Figure 3D). The γ‐lactone ring and carbon chain could, in conjunction with other biosynthetic enzymes, be the product of the highly reducing PKS (D0Z07_8189) similar to the secondary metabolite chaetoviridin.^22^ The formate moiety is unusual from a biosynthetic standpoint, but could be the result of our chromatographic purification protocol, which relies on formic acid in the mobile phase. By comparing HPLC chromatograms of a hyphodiscorubrin standard and *Hyphodiscus* crude extract under mobile phase conditions that do not contain formic acid, it is apparent that a peak for hyphodiscorubrin is not present in the crude extract. This supports the notion that the presence of the formate group may be a purification artefact (Figure S10). Furthermore, we did attempt purification of a coloured metabolite using alternate mobile phase conditions. However, we were only able to isolate a sufficient amount of material for characterization when formic acid was included in the mobile phase.

**Figure 3:**
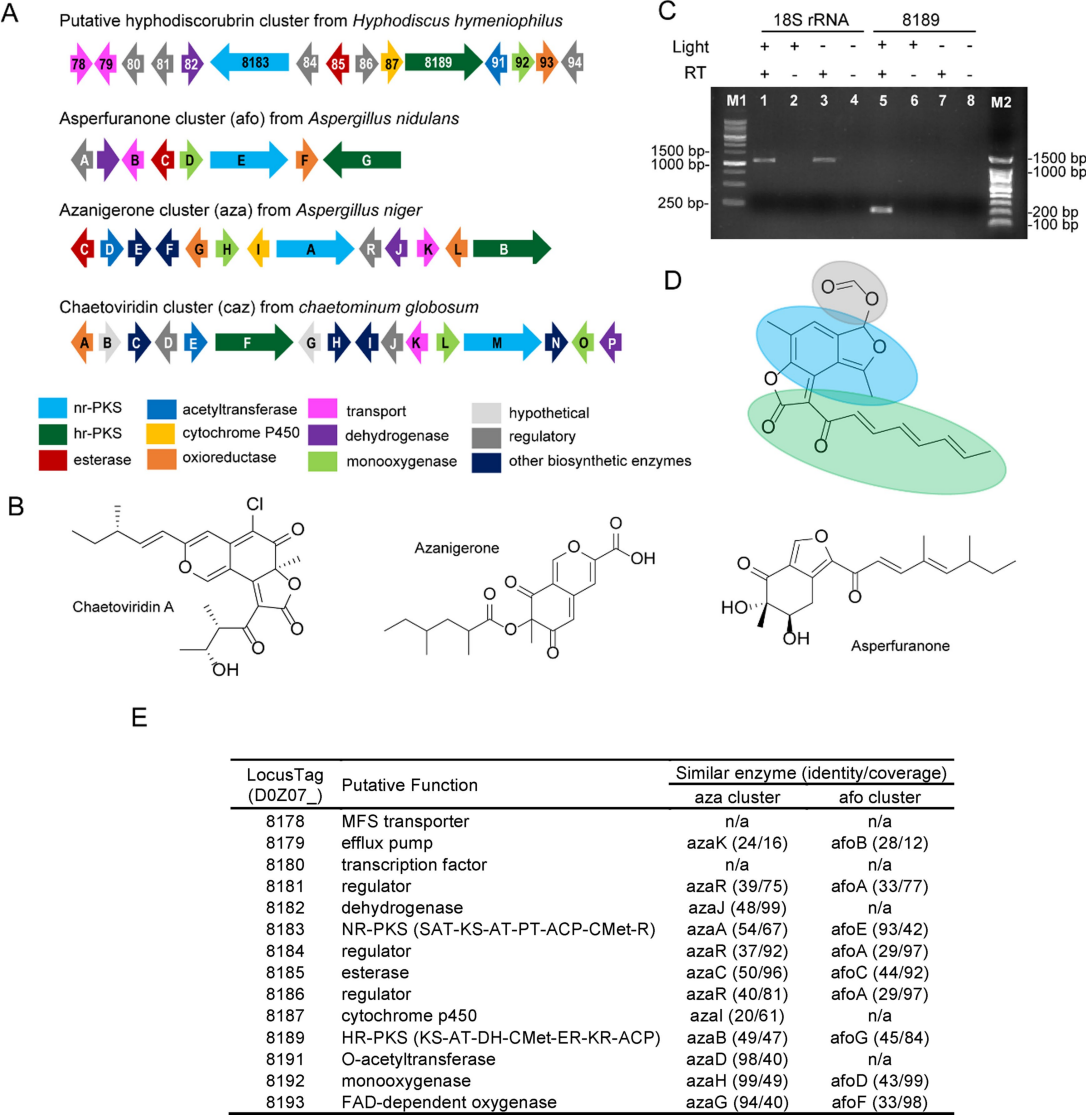
Gene cluster and structure for hyphodiscorubrin compared to other characterized natural products and gene clusters from fungi. A) Dual PKS gene cluster from *Hyphodiscus* and homologous gene clusters that have been characterized in other filamentous fungi. B) Structures of metabolites produced by the dual PKS gene clusters described in (A). C) RT‐PCR comparing the expression of a gene in the putative hyphodiscorubrin cluster (polyketide synthase D0Z07_8189) and the expression of 18S rRNA in *Hyphodiscus* under both light (+) and no light (−) conditions. NS1 and NS4 primers that amplify a portion of 18S rRNA are used as control (lanes 1–4). Primers used to amplify gene D0Z07_8189 give an expected product of 194 bp and are shown in lanes 5–8. RT (reverse transcriptase)‐negative controls are shown in lanes 2, 4, 6 and 8. RNA that was isolated from the fungal sample exposed to light was used in the reactions shown in lanes 1, 2, 5 and 6. RNA isolated from the fungal sample that was not exposed to light was used in reactions shown in lanes 3, 4, 7, and 8. M1 is a Froggabio 1 kb DNA ladder; M2 is a Froggabio 100 bp ladder. D) Proposed structure of hyphodiscorubrin with possible biosynthetic fragments from the NR‐PKS (blue), HR‐PKS (green), and from purification protocol (grey). E) Comparison of genes present in dual PKS gene cluster with genes in aza and afo clusters including predicted domains in the PKS.

To further explore the notion that this dual‐PKS cluster is indeed responsible for producing hyphodiscorubrin, we conducted reverse transcriptase PCR (RT‐PCR) on RNA isolated from *Hyphodiscus* samples grown under both light and dark conditions using primers designed to amplify a portion of the highly reducing‐PKS present in this cluster. This experiment shows that the highly reducing‐PKS gene (D0Z07_8189) is transcribed at a higher level when the fungus is exposed to light (Figure 3C) and supports that this cluster may responsible for the biosynthesis of hyphodiscorubrin.

Light is a known stimulus that has been shown to modify the production of secondary metabolites in fungi.^24^ As the production of this red molecule is influenced by light, we explored the genome near this dual PKS gene cluster to see if there were any clues about the type of light regulatory mechanism in place. Although there are several genes, including velvet genes, scattered throughout the genome which may have a global influence on the metabolome, the feature that caught our attention is a gene (D0Z07_8194) that contains a retinal domain which is adjacent to this cluster of interest. Due to the proximity of this gene to the dual PKS cluster, we imagine that this gene could feasibly have a role in regulating the light induced expression of this cluster. Further experiments would be necessary to validate the potential role of this gene in secondary metabolite regulation.

As bioactivity trials with crude extract and pure hyphodiscorubrin had no antibiotic, anti‐fungal or insecticidal activity, we presume that the role of this compound is to provide protection from sunlight. This is supported by the light activation of this secondary metabolite.

This work provides the first genome‐level insight into the *Hyphodiscus* fungal genus. We describe the natural product potential of this organism and have discovered a novel, light‐induced polyketide, hyphodiscorubrin. We have also identified a dual‐PKS gene cluster which is a candidate for the biosynthesis of hyphodiscorubrin. It is clear that the biosynthetic potential of this organism is much greater than that observed through chemical and biological analysis of extracts from laboratory‐grown cultures. As new drug leads for antibiotics, antifungals and many other applications are necessary, we suggest that new strategies be applied to try and harvest this rich chemical potential.

## Experimental Section

Experimental details can be found in the Supporting Information.

## Conflict of interest

The authors declare no conflict of interest.

## Supporting information

As a service to our authors and readers, this journal provides supporting information supplied by the authors. Such materials are peer reviewed and may be re‐organized for online delivery, but are not copy‐edited or typeset. Technical support issues arising from supporting information (other than missing files) should be addressed to the authors.

Supplementary
