# Supplementary material for: Dual‐PKS Cluster for Biosynthesis of a Light‐Induced Secondary Metabolite Found from Genome Sequencing of Hyphodiscus hymeniophilus Fungus
Source: Chembiochem. 2020 Apr 21;21(15):2116–20. doi: 10.1002/cbic.201900689 (PMC7496686; doi:10.1002/cbic.201900689)
Supplement: Supplementary file 1 — Supplementary [file CBIC-21-2116-s001.pdf]

ChemBioChem

Supporting Information

Dual-PKS Cluster for Biosynthesis of a Light-Induced Secondary Metabolite Found from Genome Sequencing of *Hyphodiscus hymeniophilus* Fungus

Glenna J. Kramer, Sheila Pimentel-Elardo, and Justin R. Nodwell© 2020 The Authors. Published by Wiley-VCH Verlag GmbH & Co. KGaA. This is an open access article under the terms of the Creative Commons Attribution License, which permits use, distribution and reproduction in any medium, provided the original work is properly cited.

Supporting Tables	Page 2
Supporting Figures	Page 3
Experimental Methods	Page 14
References	Page 17

Strain	Accession
Aspergillus niger ATCC 16888	NR_111348.1
Cladosporium cladosporioides strain DAOM 196948	JN942904.1
Monascus purpureus strain JCM 22619	JN942661.1
Aspergillus niger strain DAOM 221143	JN942866.1
Penicillium roqueforti strain DAOM 231152	JN942910.1
Emericella nidulans strain DAOM 222012	JN942873.1
Lecanora achroa strain Papong 6458	JN943714.1
Varicosporium elodeae strain AU_CRYP05	JN995640.1
Chlorociboria clavula strain D1594asc2	JN943465.1
Terfezia aff. olbiensis TLO-3	HQ698104.1
Cordyceps militaris ATCC 34165	https://www.atcc.org/products/all/34165.aspx#specifications
Trichoderma amazonicum strain IB50	HM142358.1
Candida parapsilosis strain Ph131	JN942631.1
Candida albicans strain Ph107	JN942647.1
Hyaloscypha aureliella strain M234	JN943611.1
Hyphodiscus hymeniophilius ATCC 34498	https://www.atcc.org/products/all/34498.aspx#specifications

Table S1. Strains and accession information for ITS regions used for phylogenetic tree construction.

Genome size (Mba)	35.1
Number of genes	8224
Number of exons	25564
Number of introns	17349
Total gene length (Mba)	14.2
Mean gene length (bases)	1733
Gene density (genes/Mba)	233.8
Mean exon length (bases)	497
Mean intron length (bases)	90
Mean introns per gene	2
Genome coding (%)	40.5
Predicted secondary metabolites	24
mtDNA size (ba)	61228

Table S2: Features of *Hyphodiscus* genome sequence and assembly.

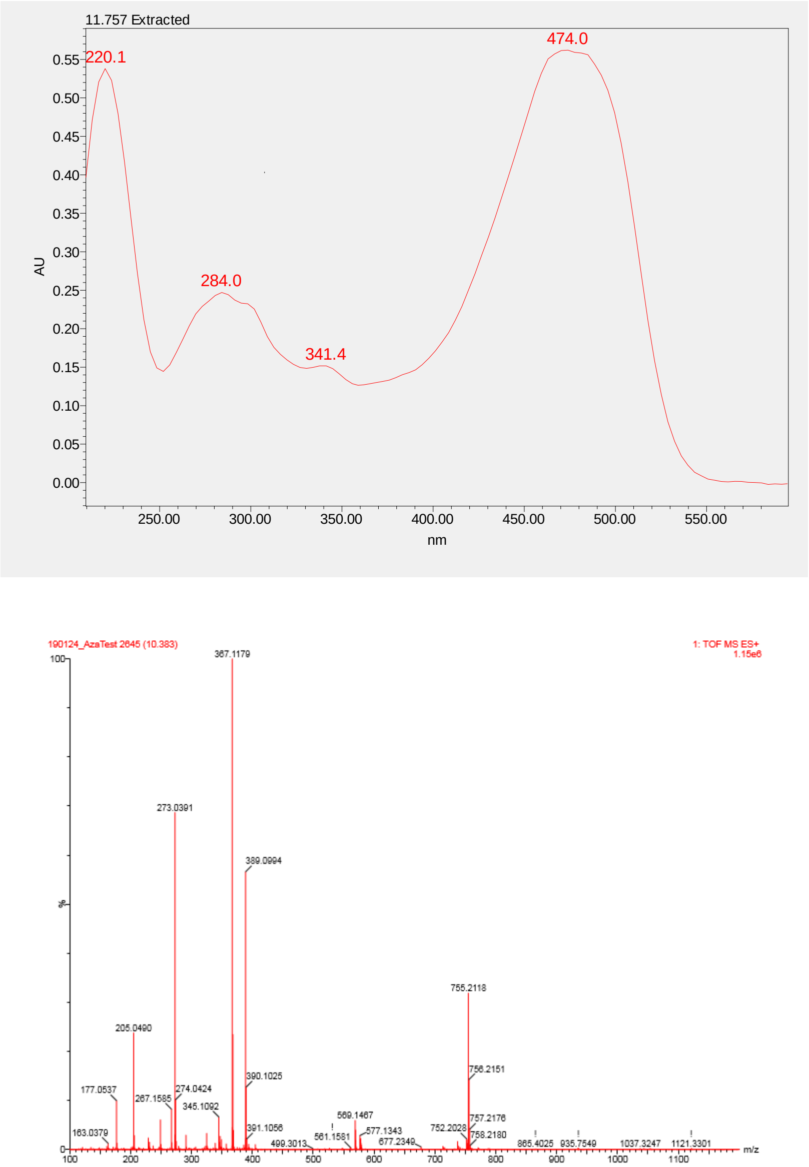

Figure S1: UV spectra and high resolution mass spectra (HRESIMS) of hyphodiscorubrin.

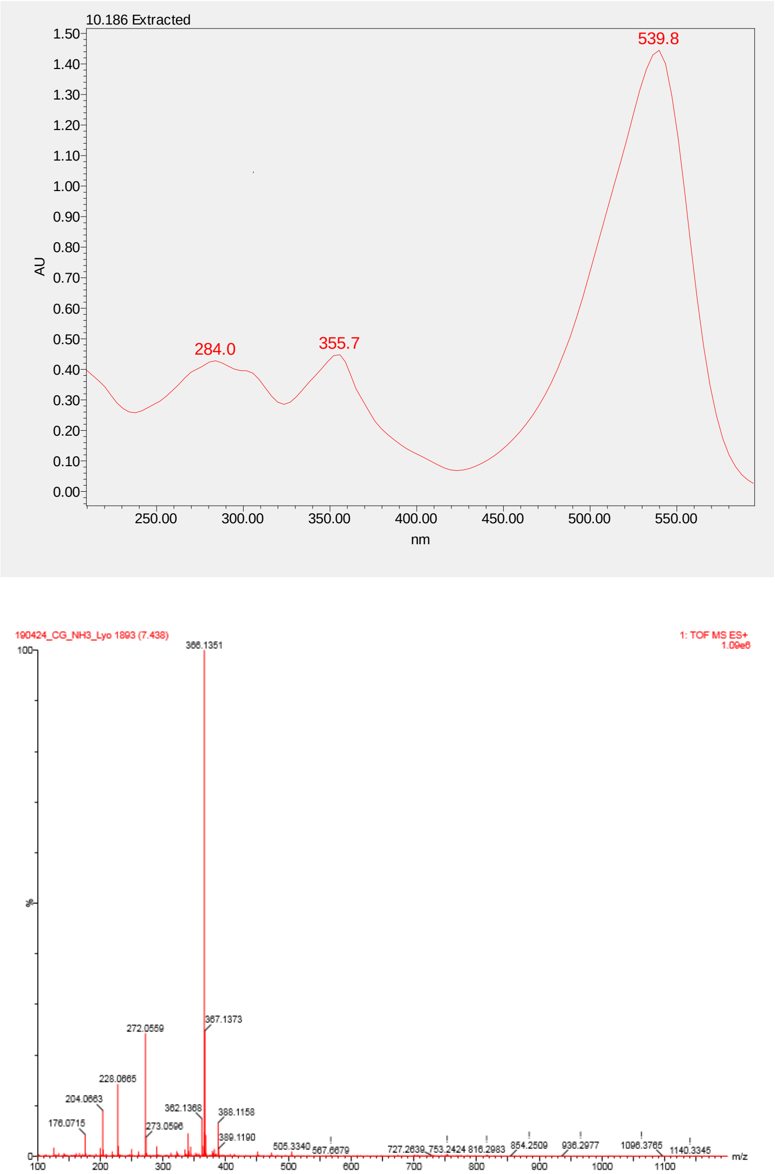

Figure S2: UV spectra and high resolution mass spectra (HRESIMS) of metabolite in an ammonium acetate buffer system.

A

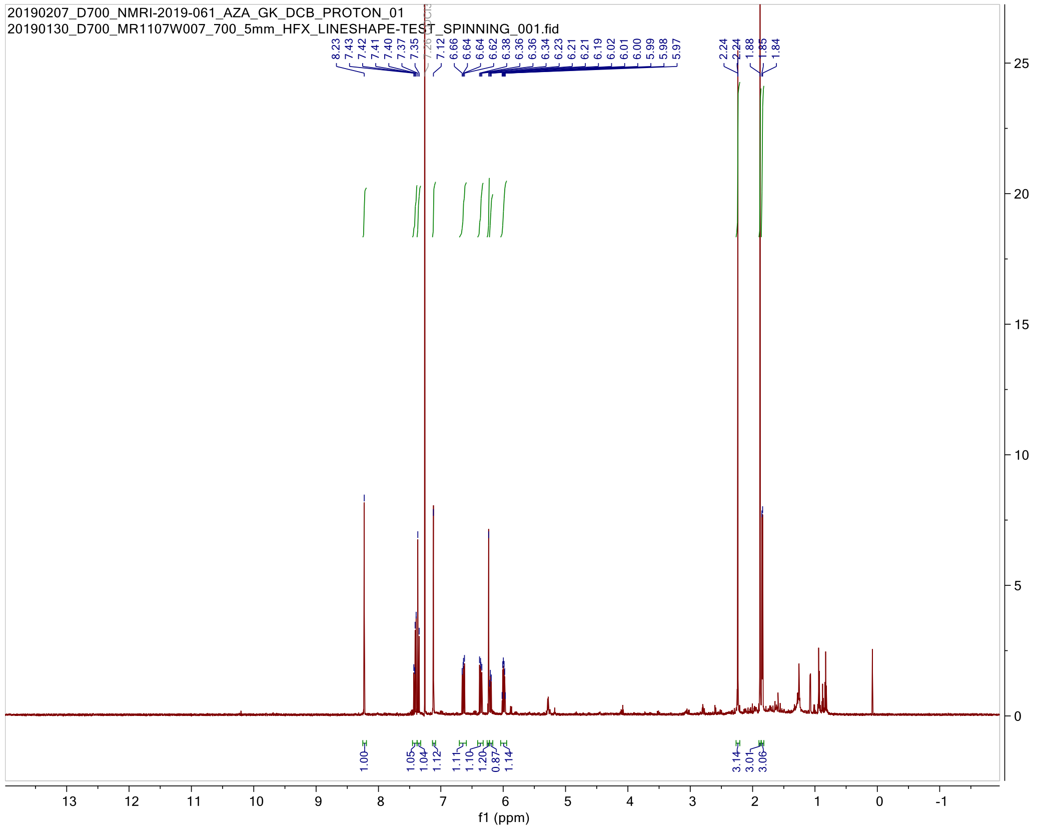
20190207_D700_NMRI-2019-061_AZA_GK_DCB_PROTON_01
20190130_D700_MR1107W007_700_5mm_HFX_LINESHAPE-TEST_SPINNING_001.fid
f1 (ppm)

B

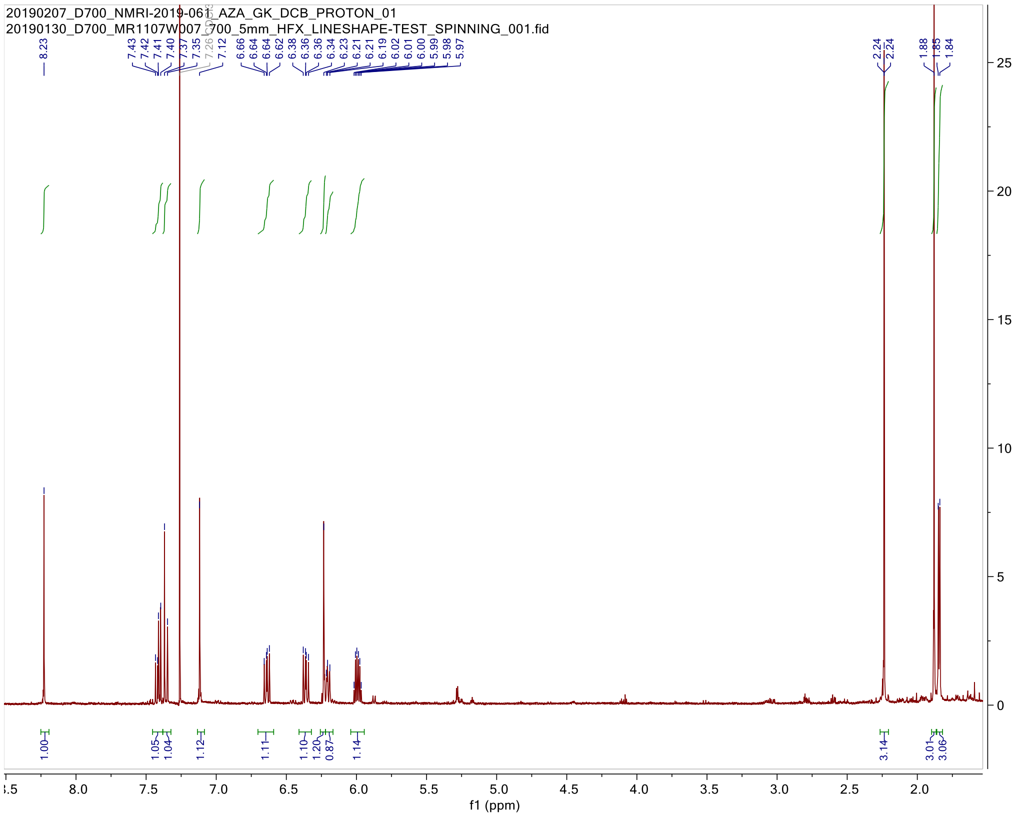
20190207_D700_NMRI-2019-061_AZA_GK_DCB_PROTON_01
20190130_D700_MR1107W007_700_5mm_HFX_LINESHAPE-TEST_SPINNING_001.fid
f1 (ppm)

C

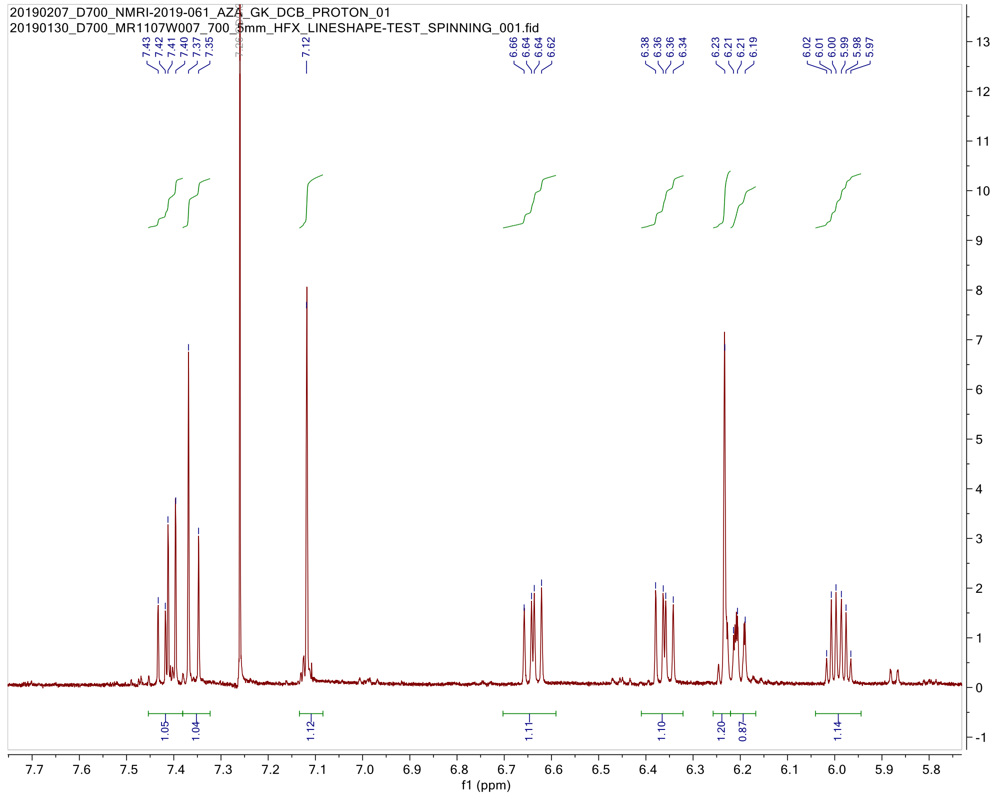

D

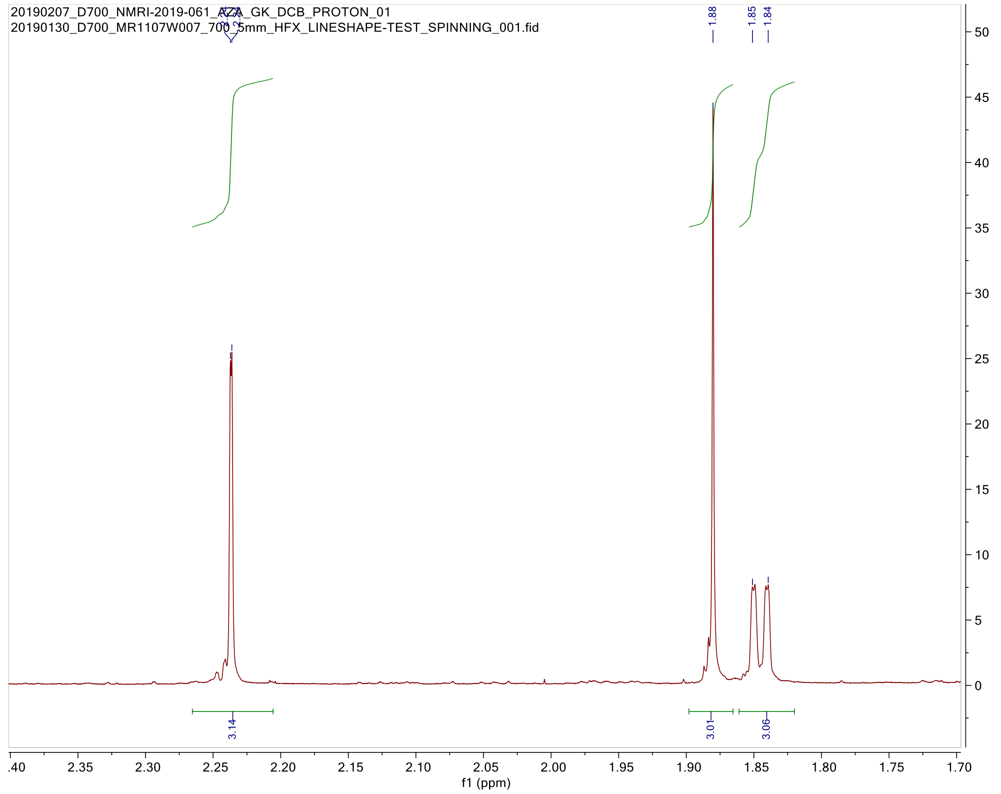

Figure S3. ^{1}H NMR (700 MHz) data of hyphodiscorubrin $CDCl_3$: A. Full spectrum. B-D. Zoom in on select ranges.

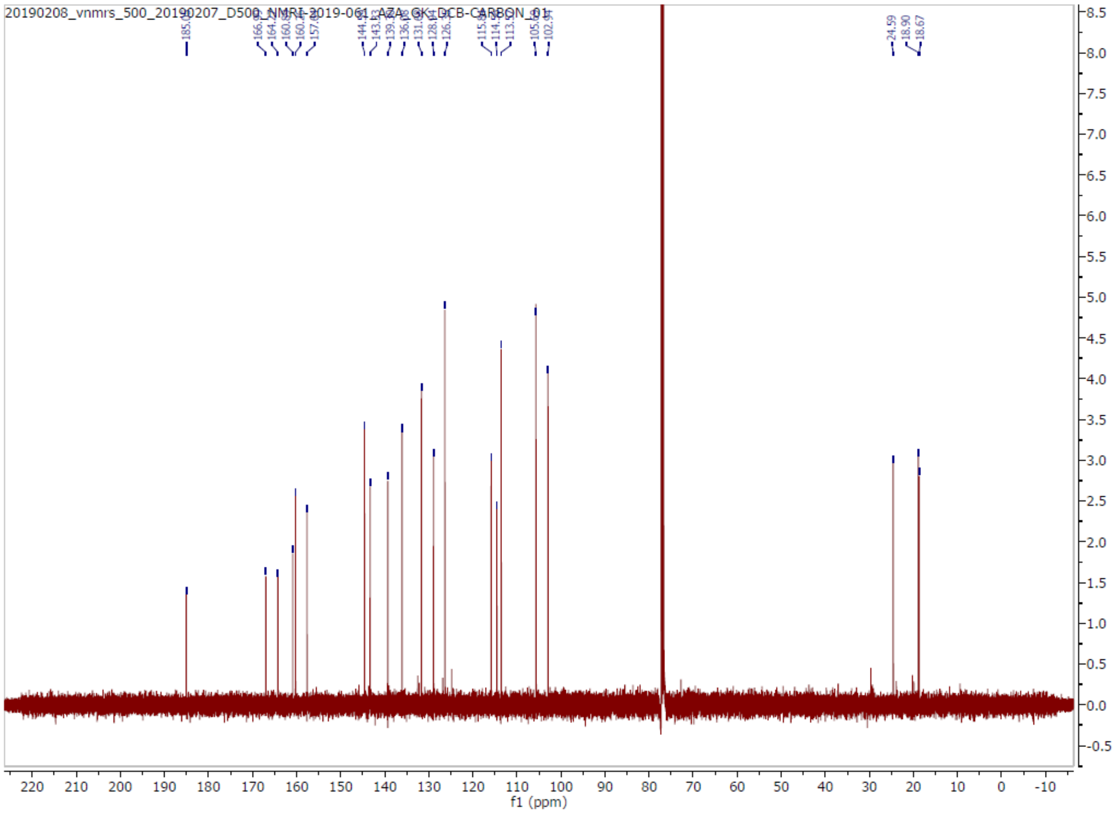

Figure S4. ^{13}C NMR (125 MHz) data of hyphodiscorubrin in $CDCl_3$

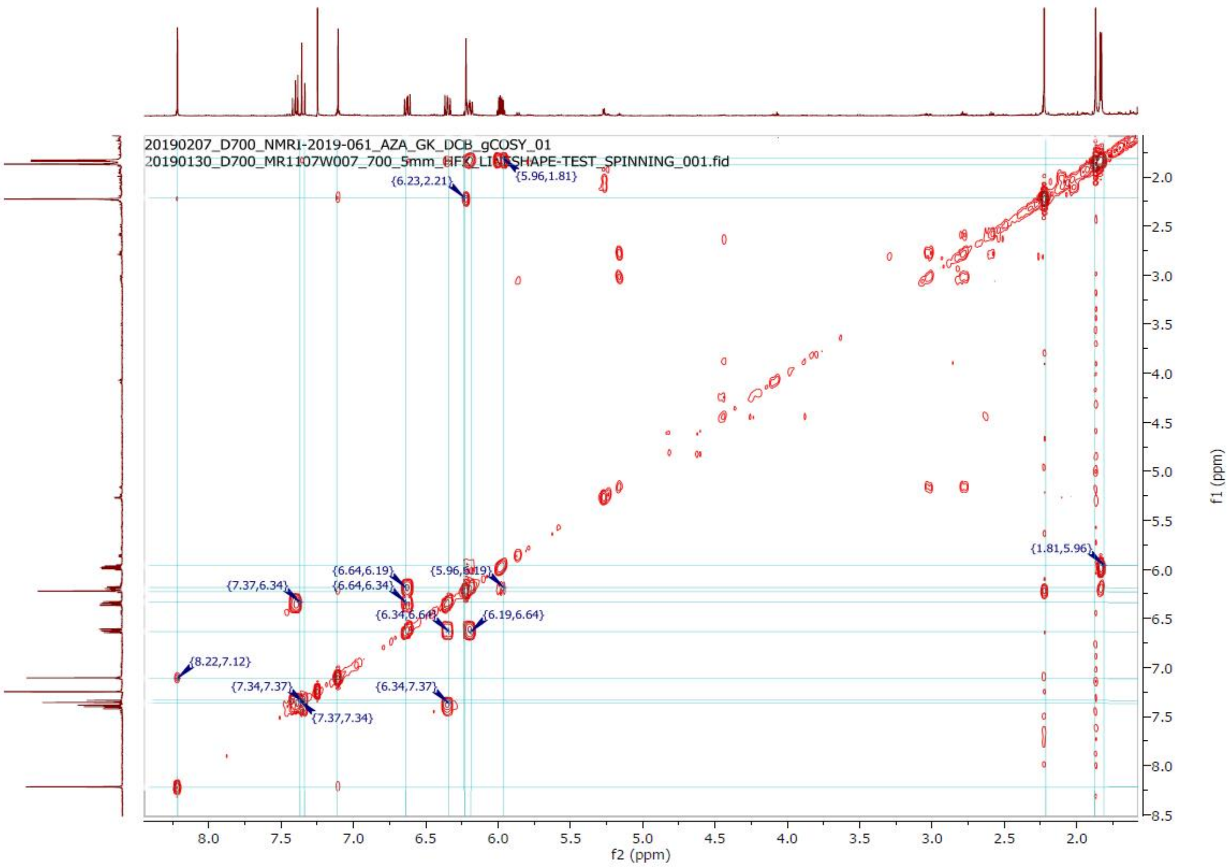

Figure S5. ^{1}H – ^{1}H COSY NMR (700 MHz) of hyphodiscorubrin in $CDCl_3$

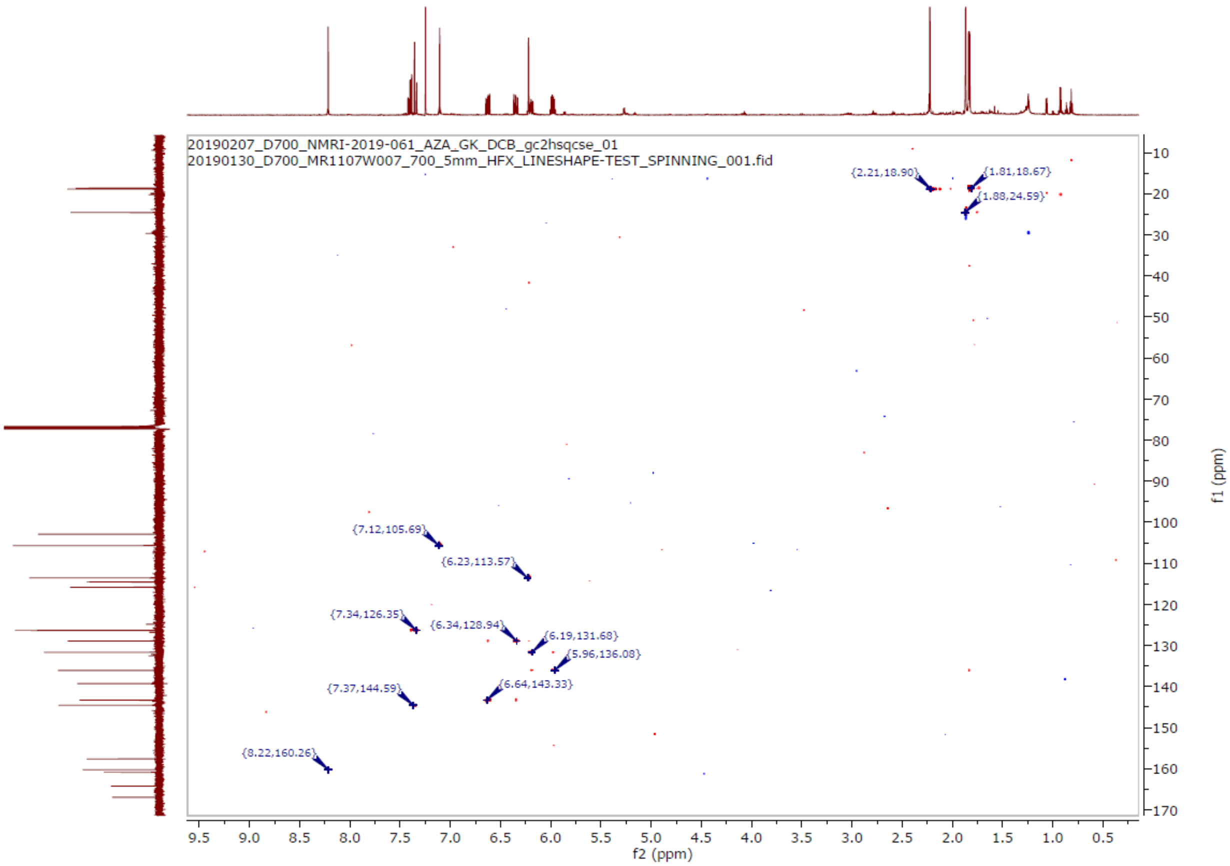

Figure S6. ^{1}H – ^{13}C HSQC-DEPT (700 MHz) of hyphodiscorubrin in $CDCl_3$

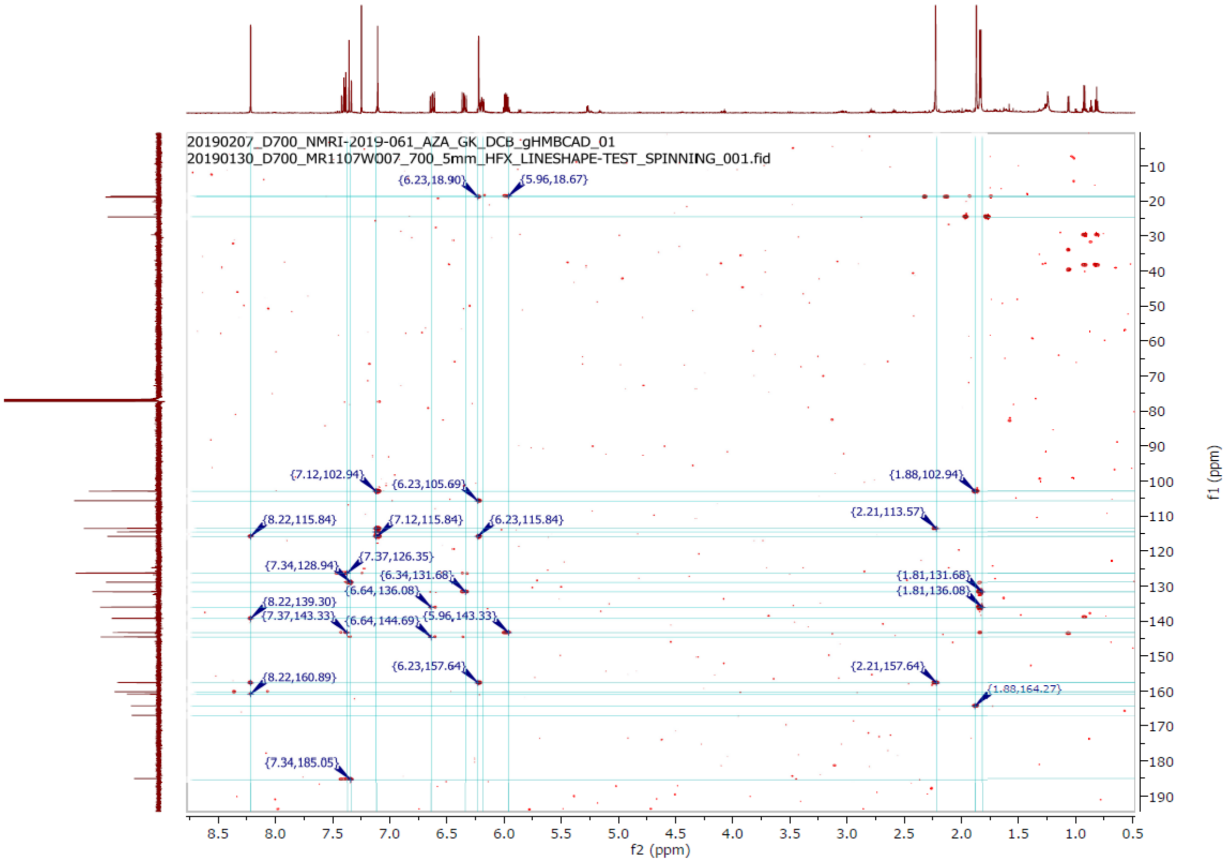

Figure S7. ^{1}H – ^{13}C HMBC (700 MHz) of hyphodiscorubrin in $CDCl_3$

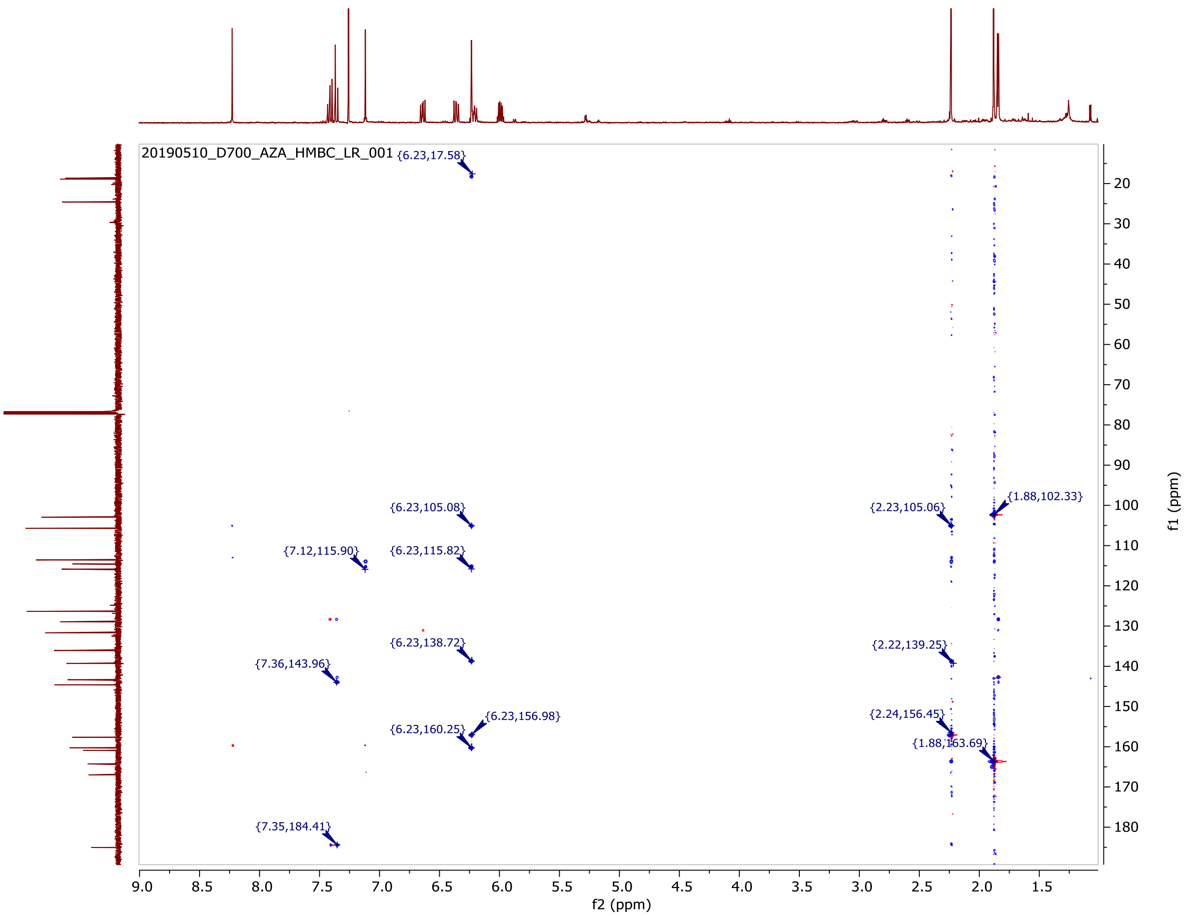

Figure S8. Long range ^{1}H – ^{13}C HMBC (700 MHz) of hyphodiscorubrin in $CDCl_3$

A.

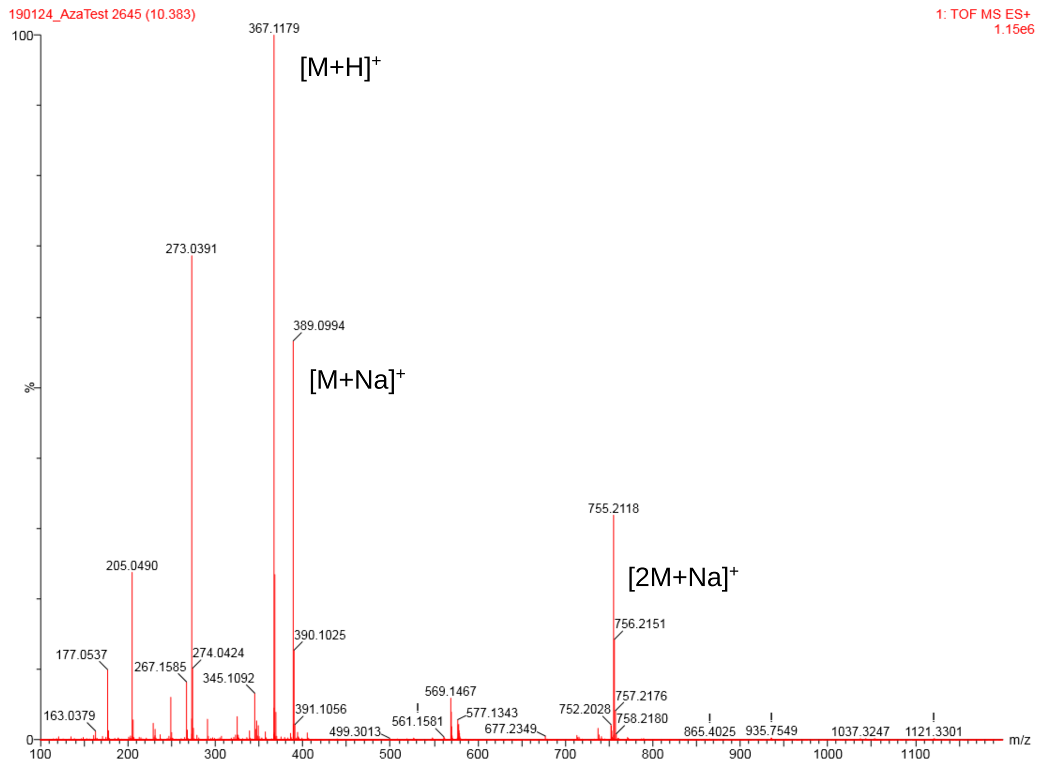

B.

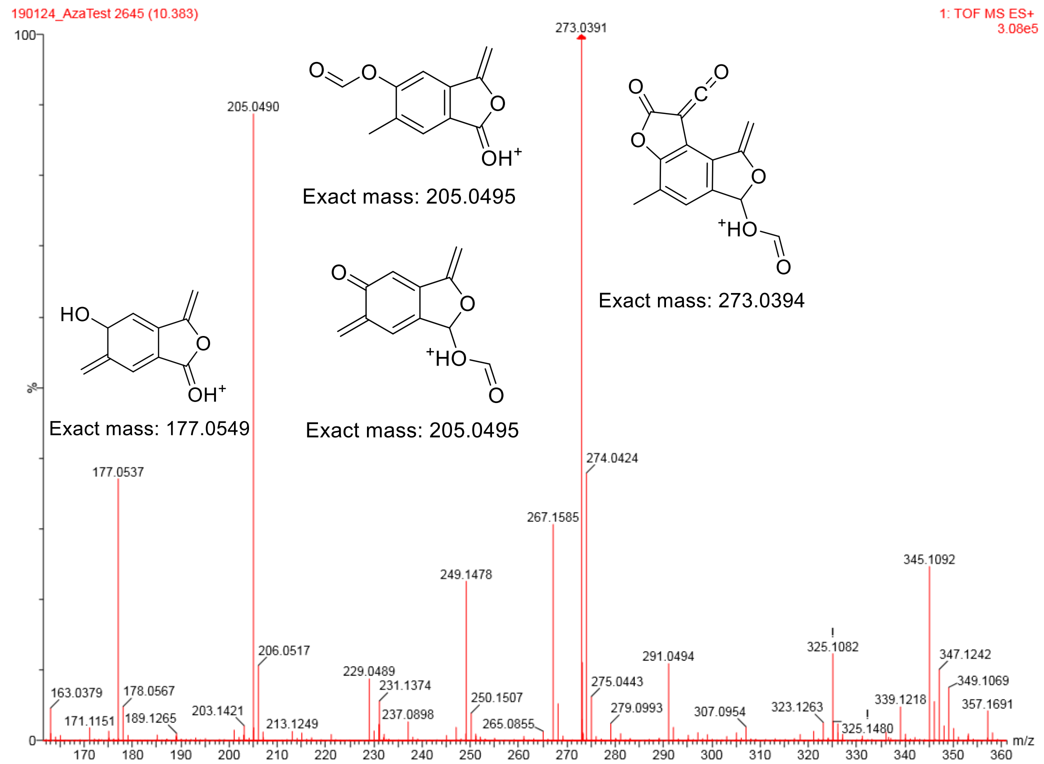

Figure S9: High resolution mass spectrometry (HRESIMS) spectra of hyphodiscorubrin. A. Full spectrum. B. Zoom in on spectrum showing source fragmentation and structures of some of the possible high abundance fragments.

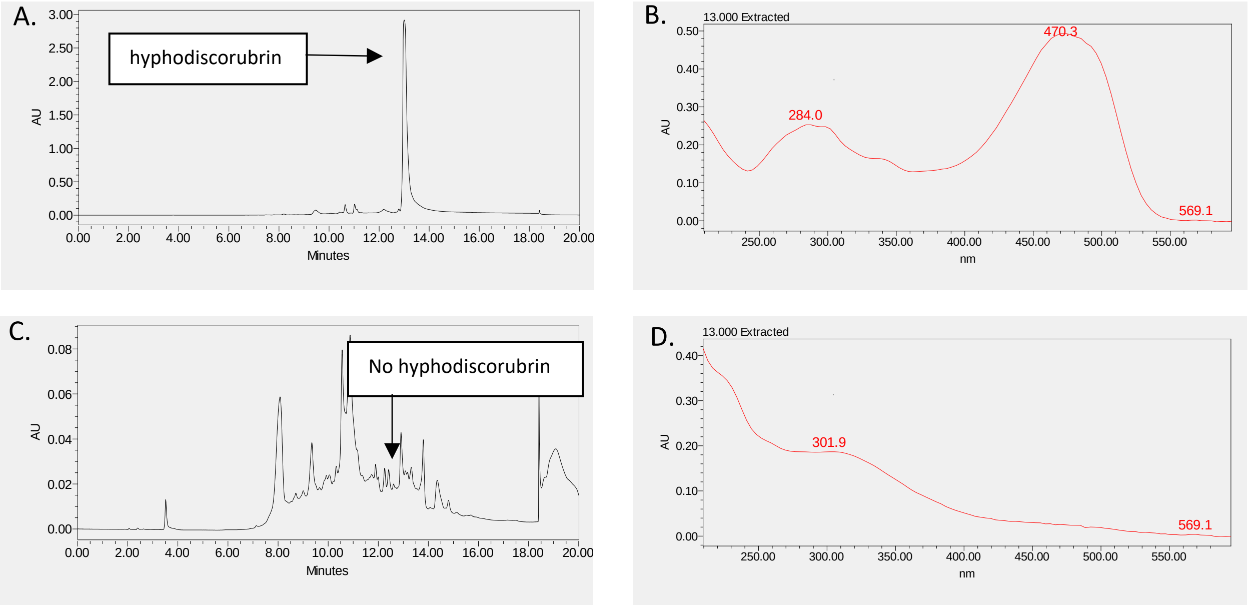

Figure S10: HPLC trace of hyphodiscorubrin standard in a water and acetonitrile buffer system compared to HPLC trace of the crude extract in a water and acetonitrile buffer system. A. Hyphodiscorubrin standard at 470 nm. B. Extracted spectrum of hyphodiscorubrin with a retention time of 13.0. C. Crude extract chromatogram at 470 nM. D. Crude extract spectrum at retention time of 13.0.

Experimental Methods

Phylogenetic tree construction

Evolutionary analysis by maximum likelihood method was performed by comparing ITS regions of several fungal species (Supporting Table 2). The evolutionary history was inferred by using the Maximum Likelihood method and Tamura-Nei model.[1] The tree with the highest log likelihood (-6011.76) is shown. Initial tree(s) for the heuristic search were obtained automatically by applying Neighbor-Join and BioNJ algorithms to a matrix of pairwise distances estimated using the Maximum Composite Likelihood (MCL) approach, and then selecting the topology with superior log likelihood value. The tree is drawn to scale, with branch lengths measured in the number of substitutions per site. This analysis involved 16 nucleotide sequences. Codon positions included were 1st+2nd+3rd+Noncoding. There were a total of 735 positions in the final dataset. Evolutionary analyses were conducted in MEGA X.[2]

Strain and maintenance

Hyphodiscus hymeniophilus ATCC® 34498 was received from the American Type Culture Collection (ATCC). Fungal cultures were maintained at 23.0 °C on potato dextrose agar.

Genomic DNA extraction and purification

Genomic DNA was extracted and purified as described in Kramer and Nodwell.[3] Culture tube containing 5 mL of liquid seed media (10g peptone, 40g maltose, 10g yeast extract, 1g agar in 1L DI water) were aseptically inoculated with a 3mm square agar slab containing mycelial growth. The fungal mycelia were grown for 1 week at 23.0°C. The mycelia were harvested, rinsed with sterile TE buffer and then frozen in liquid nitrogen. The frozen material was macerated with a mortar and pestle. The resulting powder was transferred to a set of epitubes and 500µL of CTAB DNA extraction buffer was added (100mM Tris pH = 8.0, 10 mM EDTA, 2% CTAB, 2.8M NaCl). After an incubation at room temperature for 5min, 2µL of RNAse A (Thermo Scientific, 10mg/mL) and 10µL of Proteinase K were added (Invitrogen, 10mg/mL) and the tubes were inverted to mix. After centrifuging for 5min, the pellet was ground in the epitube with a pellet pestle, then incubated for an additional 5min before purification with phenol-chloroform. Each sample was washed twice with phenol-chloroform (50:50, phenol buffered with Tris pH 8.0, 600µL) then twice with chloroform (600 µL). The resulting DNA containing aqueous portions were pooled and DNA was precipitated using cold ethanol (2.5× sample volume) and 3M sodium acetate (0.1× sample volume). DNA was precipitated for at least 30 min by storing at −20°C. The DNA precipitate was collected by centrifuging for 30min, the pellet was washed with 70% ethanol and resuspended in TE buffer. The DNA was further purified with AMPure XP beads (Agencourt) by using an equal volume of beads to volume of DNA and eluting into TE. DNA was quantified using a PicoGreen assay (ThermoFisher) prior to sequencing.

Genome sequencing and assembly

The purified DNA was sequenced using Pacific Biosciences RS II sequencing at the Genome Quebec Innovation Center (McGill University, Montreal, Canada). The sample was prepared using a large insert sheared DNA library and was sufficient for sequencing 8 SMRT cells. The first assembler chosen was the PBcR pipeline from the Celera assembler (version 8.3rc2) using input genome sizes of 25, 30, 35, 40 and 45 Mba.[4] The second assembler was SMRT portal (version 2.3.0) launched from an Amazon machine image. Assembly was performed on all 8 SMRT cells using the RS_HGAP Assembly.2 application with default settings and a genome size of 35 Mbp.[5] The resulting assembly yielded 25 contigs, with 19 of these contigs contained telomeres on both ends of the contig and three contigs containing telomeric repeats on one end. The other three remaining contigs consisted of a mitochondrial DNA sequence, the PacBio internal standard and a collapsed non-ribosomal DNA region with high sequence coverage that was identical to the non-ribosomal DNA region contained in one of the other contigs. After removing the internal

standard and the extra non-ribosomal DNA, the resulting DNA sequence consisted of 23 contigs: 22 contigs of genomic DNA and one contig of mitochondrial DNA (mtDNA).

Annotation of genome and identification of secondary metabolite gene clusters
Genome annotation was performed using the MAKER (version 2.31.8) pipeline and secondary metabolite gene clusters were predicted using FungiSMASH (version 5.0). Further experimental details are given in supporting information using three *ab inito* gene prediction methods: Augustus trained for *Fusarium graminearum*, and GeneMark-ES and SNAP self-trained on the *Hyphodiscus* genome.[6–9] Protein data and EST data from Leotinomyces, which were compiled from the NCBI database, was used as protein and EST evidence in MAKER. Repeat elements were identified using Repeat Masker using the Repbase Library 20150807. A final set of consensus gene predictions was chosen using Exonerate.[10] The gene models were functionally annotated using the BLAST component of the Blast2GO software package and searching against the SwissProt protein database (accessed July 2018) with the best hit being selected.[11] Secondary metabolite genes and gene clusters were predicted using FungiSMASH version 5.0.[12]

Production and Extraction of Red Metabolite
Culture tubes containing 5 mL of liquid seed media (10g peptone, 40g maltose, 10g yeast extract, 1g agar in 1L DI water) were aseptically inoculated with a 3mm square agar slab containing mycelial growth. The fungal mycelia were grown for 1 week at 23.0°C. This liquid seed culture was used to inoculate a petri dish of potato dextrose agar (PDA) by spreading 1 mL of culture on the plate. *Hyphodiscus* was grown at 23ºC under dark conditions for 2 weeks. After 2 weeks the plates were exposed 12-hour light/dark cycles of broad spectrum light for 4 days to turn on production of red pigment. Red pigment was extracted three times using ethyl acetate by covering macerated agar and fungal material with the solvent, allowing the material to soak for 10 minutes, then decanting off the solvent. The extracts were pooled and then dried using a rotary evaporator.

Purification of red molecule
The red molecule was purified from the *Hyphodiscus* extract using two rounds of HPLC. First, the crude extract was purified on a semi-preparative HPLC column (Luna C18, 100A, 250 x 10.00 mm, 5 micron) using a flow rate of 5 mL/min over a gradient from 95% buffer A (water with 0.1% formic acid) in buffer B (acetonitrile with 0.1% formic acid) to 5% buffer A in buffer B for 10 minutes then by holding 5% buffer A in buffer B for 5 minutes on an Alliance 2695 HPLC series (Waters, USA). The orange-red molecule that was eluted at a retention time of 11.5 minutes was collected and dried down using a rotary evaporator. Final purification was carried out using an analytical HPLC column (Luna C18, 100A, 250 x 4.60 mm, 5 micron) with a flow rate of 1 mL/min over a gradient from 95% buffer A (water with 0.1% formic acid) in buffer B (acetonitrile with 0.1% formic acid) to 5% buffer A in buffer B for 15 minutes then holding 5% buffer A in buffer B for 5 minutes. The purified molecule was collected with a retention time of 16.5 minutes and UV maximums of 284nm, 341nm and 470nm, was dried using a rotary evaporator then lyophilized. Extraction of mycelial growth from 24, 10 cm petri dishes would yield approximately 2 mg of the purified metabolite.

Bioactivity Screens
Crude and semi-purified extracts were tested for antibiotic or antifungal against *E. coli¸ B. subtilis,* and *S. cerevisiae* activity using a disk diffusion approach. Specifically, 10 µL of a 1 mg/mL re-suspended extract solution was soaked onto a sterile disk, which was placed on a petri dish that was freshly spread with a seed culture of the microbe that was being tested against. Susceptibility of *D. melanogaster* against these extracts was tested by placing 1st instar larvae in a tube with dried food which was reconstituted using water containing 100 µg/mL of the extract. The larvae were observed over a 2-week period for variations in growth and development that differed from the control sample, which contained no small molecule extract.

MS and NMR analysis

UPLC-MS was carried out using an Acquity UPLC system coupled to a Xevo G2-S QTof (Waters, USA) on an Acquity UPLC BEH C18 column (2.1 x 50 mm) using a gradient of 95% buffer A (water with 0.1% formic acid) in 5% buffer B (acetonitrile with 0.1% formic acid) to 5% buffer A in 95% buffer B over 15 minutes. The peak corresponding to our molecule of interest eluted at 11.5 minutes and had relevant source fragmentation. The MS conditions for positive mode were set to a capillary voltage of 2.5 kV, a desolvation gas flow of 500 L/h (N_2) and a source temperature of 120°C. All NMR data was acquired in $CDCl_3$ on a 700 MHz Agilent DD2 NMR Spectrometer with the exception of the ^{13}C NMR data, which was acquired on a 500 MHz Agilent DD2 NMR Spectrometer with a cryogenically cooled probe optimized for ^{13}C NMR acquisition.

RNA extraction

Culture tubes containing 5 mL of liquid seed media (10g peptone, 40g maltose, 10g yeast extract, 1g agar in 1L DI water) were aseptically inoculated with a 3mm square agar slab containing mycelial growth. The fungal mycelia were grown for 1 week at 23.0°C. The mycelia were used to inoculate potato dextrose agar covered in sterile cellophane. The fungi were grown in the dark at room temperature on the plates for 10 days, after which half of the plates were exposed to light and the other half remained in the dark. After 3 days, the fungi were harvested under low light conditions that limited exposure to RNAses. The fungal mycelia, from both light exposed and dark growth conditions, were scraped off of the cellophane, frozen in liquid nitrogen and crushed with a mortar and pestle. The crushed mycelia from one 60 mm petri dish was divided between 4 epitubes with 500 µL TRIzol reagent and extracted with 500 µL of chloroform: isoamyl alcohol (24:1) twice. The aqueous layer was retained and the nucleic acids were precipitated by using cold ethanol (2.5× sample volume) and 3M sodium acetate (0.1× sample volume). After centrifugation, the pelleted nucleic acids were resuspended in 5x DNAse buffer and RNAse-free water (500 µL total volume) and treated with DNAse (10 µL) for 1 hour at 37°C. The DNAse was removed and the RNA further purified by washing each sample twice with phenol-chloroform (50:50, phenol buffered with Tris pH 8.0, 600µL) then twice with chloroform (600 µL). The resulting RNA containing aqueous portions were pooled and RNA was precipitated using cold ethanol (2.5× sample volume) and 3M sodium acetate (0.1× sample volume). RNA was precipitated for at least 2 hours by storing at −20°C. The RNA was collected by centrifuging for 10 min and the pellet was washed with 70% ethanol and resuspended in RNAse-free water. The integrity of the RNA was accessed through gel chromatography and the 260/280 ratio. The concentration of RNA was also calculated. All materials used in the RNA extraction process were either ordered as RNAse free, treated with RNAse Zapper or autoclaved twice to prevent sample exposure to RNAses.

RT-PCR

RT-PCR was performed using a QIAGEN OneStep RT-PCR kit, using manufacturer instructions by combining RNAse-free water, QIAGEN OneStep RT-PCR Buffer, dNTP (400 µM final concentration of each dNTP), forward primer (0.6 µM final concentration), reverse primer (0.6 µM final concentration), QIAGEN OneStep RT-PCR Enzyme Mix and template RNA (1 ng/ µL final concentration). The primer pair to detect the PKS 8189 was 8189_For 5'- GGTATTTGTACGCGCCTGAT-3' and 8189_Rev 5'-GGCGTTGTAGATGGACTGGT-3' and is expected to give a 194 bp product. The control primers used to detect 18S rRNA were the broadly used NS1 and NS4 primer pair.[13] A touchdown PCR program was used. There was an initial reverse transcription step for 30 minutes at 50°C. This was followed by an initial PCR activation step for 15 minutes at 95°C. Then, the three step touchdown cycling went as such: denaturation (95°C, 30 seconds), annealing (30 seconds), extension (72°C, 1.5 minutes). Every three cycles the annealing temperature decreased from 63°C to 59°C, 55°C, and 51°C. Then there were 15 cycles of denaturation (95°C, 30 seconds), annealing (46°C, 30 seconds), and extension (72°C, 1.5 minutes). This was followed by a final extension for 10 minutes at 72°C. All reactions were kept on ice until a hot start. The reverse transcriptase (RT) negative control was performed by skipping the reverse transcription step

and placing the reaction into the thermal cycler at the beginning of the initial PCR activation step. The product (5 µL) was combined with loading dye and run on a 1% agarose TAE gel containing RedSafe and visualized under UV.

References

(1) Tamura, K.; Nei, M. Estimation of the Number of Nucleotide Substitutions in the Control Region of Mitochondrial DNA in Humans and Chimpanzees. *Mol. Biol. Evol.* **1993**, *10* (3), 512–526. https://doi.org/10.1093/oxfordjournals.molbev.a040023.

(2) Tsai, I. J.; Zarowiecki, M.; Holroyd, N.; Garciarrubio, A.; Sanchez-Flores, A.; Brooks, K. L.; Tracey, A.; Bobes, R. J.; Fragoso, G.; Sciutto, E.; et al. The Genomes of Four Tapeworm Species Reveal Adaptations to Parasitism. *Nature* **2013**, *496* (7443), 57–63. https://doi.org/10.1038/nature12031.

(3) Kramer, G. J.; Nodwell, J. R. Chromosome Level Assembly and Secondary Metabolite Potential of the Parasitic Fungus Cordyceps Militaris. *BMC Genomics* **2017**, *18* (1), 912. https://doi.org/10.1186/s12864-017-4307-0.

(4) Berlin, K.; Koren, S.; Chin, C.-S.; Drake, J. P.; Landolin, J. M.; Phillippy, A. M. Assembling Large Genomes with Single-Molecule Sequencing and Locality-Sensitive Hashing. *Nat. Biotechnol.* **2015**, *33* (6), 623–630. https://doi.org/10.1038/nbt.3238.

(5) Chin, C.-S.; Alexander, D. H.; Marks, P.; Klammer, A. A.; Drake, J.; Heiner, C.; Clum, A.; Copeland, A.; Huddleston, J.; Eichler, E. E.; et al. Nonhybrid, Finished Microbial Genome Assemblies from Long-Read SMRT Sequencing Data. *Nat. Methods* **2013**, *10* (6), 563–569. https://doi.org/10.1038/nmeth.2474.

(6) Cantarel, B. L.; Korf, I.; Robb, S. M. C.; Parra, G.; Ross, E.; Moore, B.; Holt, C.; Sánchez Alvarado, A.; Yandell, M. MAKER: An Easy-to-Use Annotation Pipeline Designed for Emerging Model Organism Genomes. *Genome Res.* **2008**, *18* (1), 188–196. https://doi.org/10.1101/gr.6743907.

(7) Campbell, M. S.; Holt, C.; Moore, B.; Yandell, M. Genome Annotation and Curation Using MAKER and MAKER-P. In *Current Protocols in Bioinformatics*; John Wiley & Sons, Inc.: Hoboken, NJ, USA, 2014; Vol. 48, pp 4.11.1-4.11.39. https://doi.org/10.1002/0471250953.bi0411s48.

(8) Stanke, M.; Keller, O.; Gunduz, I.; Hayes, A.; Waack, S.; Morgenstern, B. AUGUSTUS: Ab Initio Prediction of Alternative Transcripts. *Nucleic Acids Res.* **2006**, *34* (Web Server), W435–W439. https://doi.org/10.1093/nar/gkl200.

(9) Borodovsky, M.; Lomsadze, A. Eukaryotic Gene Prediction Using GeneMark.Hmm-E and GeneMark-ES. In *Current Protocols in Bioinformatics*; John Wiley & Sons, Inc.: Hoboken, NJ, USA, 2011; Vol. Chapter 4, p Unit 4.6.1-10. https://doi.org/10.1002/0471250953.bi0406s35.

(10) Slater, G. S. C.; Birney, E. Automated Generation of Heuristics for Biological Sequence Comparison. *BMC Bioinformatics* **2005**, *6* (1), 31. https://doi.org/10.1186/1471-2105-6-31.

(11) O'Leary, N. A.; Wright, M. W.; Brister, J. R.; Ciufo, S.; Haddad, D.; McVeigh, R.; Rajput, B.; Robbertse, B.; Smith-White, B.; Ako-Adjei, D.; et al. Reference Sequence (RefSeq) Database at NCBI: Current Status, Taxonomic Expansion, and Functional Annotation. *Nucleic Acids Res.* **2016**, *44* (D1), D733–D745. https://doi.org/10.1093/nar/gkv1189.

(12) Weber, T.; Blin, K.; Duddela, S.; Krug, D.; Kim, H. U.; Bruccoleri, R.; Lee, S. Y.; Fischbach, M. A.; Muller, R.; Wohlleben, W.; et al. AntiSMASH 3.0--a Comprehensive Resource for the Genome Mining of Biosynthetic Gene Clusters. *Nucleic Acids Res.* **2015**, *43* (W1), W237-43. https://doi.org/10.1093/nar/gkv437.

(13) Raja, H. A.; Miller, A. N.; Pearce, C. J.; Oberlies, N. H. Fungal Identification Using Molecular Tools: A Primer for the Natural Products Research Community. *J. Nat. Prod.* **2017**, *80* (3), 756–770. https://doi.org/10.1021/acs.jnatprod.6b01085.
